# Physiological Albuminuria

**Published:** 1905-06-24

**Authors:** 


### Hospital Clinics.

### PHYSIOLOGICAL ALBUMINURIA.

The significance of albuminuria in apparently healthy young adults who, apart from the condition of the urine, give no sign of organic disease, is a staple problem for the profession, and especially for those engaged in examinations for life assurance. Dr. Samuel West[1] in discussing the clinical bearings of this problem is able to do so in the light of a wide experience. He divides albuminuria into three main varieties, as indicated by the following table:

| | | |
|---|---|---|
| 1. Cis- or pre-renal... | Cause obvious, *e.g.* morbus cordis, fevers, etc. | |
| | Cause not obvious. | Physiological |
| 2. Renal ............... | No obvious renal disease | Physiological |
| | Obvious renal disease | |
| 3. Citra- or post-renal... | Accidental, from urinary or genital passages, etc. | |

Physiological albuminuria means no more therefore than an albuminuria whose cause is not obvious, and Dr. West very reasonably objects to the practice of dubbing as physiological a condition which is not the normal for the generality of individuals simply on the ground that no cause for it can, in a particular case, be discovered. He considers that it would be wiser and more scientific to discard the many adjectives applied to the variety of albuminuria under notice, such as intermittent, cyclical, postural or dietetic, and to call it "albuminuria in the apparently healthy," which, at all events, is plain and honest.

One of the fundamental difficulties in the matter of albuminuria is to define the meaning of the word: it is not enough to accept it, so to speak, at its face value, for, as Dr. West points out, it is possible by the application of delicate tests after concentration, to demonstrate the presence of minute quantities of albumin in any sample of urine, even though it appears albumin-free to ordinary reagents. This albumin no doubt is derived from the desquamation of cells from the mucous membrane of the urinary passages, which is a constant feature in the life-history of all such structures. We have therefore to credit the word albuminuria with a quantitative significance—a fact which, of course, introduces a margin for difference of opinion, especially in border-line cases where the demonstrable quantity of albumin is very small. But in common parlance albuminuria signifies the presence of albumin in such quantities as are capable of demonstration in unconcentrated urines with the usual clinical tests, as nitric acid, or heat with acetic acid, though the activity of these two methods in demonstrating albumin is not by any means equal. Acetic acid applied to a urine which has been boiled will sometimes show a faint clouding of the liquid which to nitric acid in the cold may appear quite free from albumin. None the less the above lax definition of albuminuria must stand for want of a better, though it is plain to how large an extent the definition involves questions not of fact but of degree.

Statistics regarding the frequency of albuminuria in the apparently healthy vary within wide limits—variations depending largely, no doubt, upon the degree of care exercised in the examination. In a long series of observations made for Dr. West a percentage of 42 was obtained. This investigation revealed the following facts. That of healthy persons nearly 60 per cent. showed no albumin at all, while of the remaining 40 in nearly a half the albumin present was not serum-albumin, but nucleo-albumin, and that in the minutest quantities. Further, of the 20 in whom the albumin was serum-albumin, one-half showed a trace so minute that only the most rigorous observation was able to determine its presence. As regards the frequency of albuminuria in the apparently healthy at different ages, it appears that some 10 per cent. of healthy infants reveal the condition (though Dr. West does not give any authority for the statement), that the percentage stands at its lowest at about the age of 25, and from that period advances regularly and rapidly with age.

The author holds that the fact of an albuminuria being intermittent is no proof that the kidneys are sound, since a patient convalescent from an attack of acute Bright's disease, who has no albuminuria while recumbent, may evince a recurrence when he gets to his feet again, and may indeed faithfully reproduce all the features currently associated with so-called physiological albuminuria. Here, none the less, it must be assumed that the kidneys, though mending, are not yet functionally restored, for with the passage of time the albumin may entirely disappear. Again, the fact that albumin is present only in minute quantity is no argument against an organic lesion of the kidneys, for it is common knowledge that advanced cirrhosis of the kidneys may be attended by insignificant albuminuria.

The age at which the condition has been most frequently studied is that ranging from the 15th to the 25th year, that is to say in boys at school, and in young adult candidates for public services or proposers for life assurance. In the case of boys at school the condition of the urine raises important questions as to whether the school-life of the boy should be interfered with. "Unless there are special reasons in a particular case, careful consideration and the balancing of pros and cons generally end in leaving the boy at school under the careful and constant supervision of his medical attendant. The risk has to be run by the boy, and, fortunately, in most cases the risk proves to be slight and the result is satisfactory."

As regards the case of young adults affected with this form of albuminuria actuarial tables compiled from the data of assurance offices show that the condition gives a definite increase to the risk. Thus, given two males, aged 36, one albuminuric and the other healthy, the expectation of death in the following year is as 3·33 to 1·16 for the two respectively. These facts raise the grave question of the relation between these cases and granular kidney. Dr. West believes the relation to be a close one, for the curves illustrating the frequency of occurrence in each at successive ages are approximately parallel. Further, in granular kidney the albumin in the urine may vary greatly in amount from time to time, and may be altogether absent for long periods, while the presence of renal casts is not a constant feature of the disease. Again, granular kidney is a very insidious disease, and though its highest mortality is at middle life, it is certain that the condition has its inception many years before it gives rise to such symptoms as will lead to its detection. "If these considerations be now applied to life-insurance we must conclude that albuminuria —*i.e.* the so-called functional albuminuria—introduces an appreciably increased risk to the life, and that this risk increases rapidly with age. The rules which have been laid down are sound and fully justified—namely, to reject all such lives above the age of 40 years, to load them heavily between 30 and 40 years, to make some addition to every case under 30 years, and to wait awhile and watch the cases in adolescence and youth. To which general rules may be added the still more important one, to reject at once at every age those in which the albuminuria is associated with arterial thickening."

The following are the author's conclusions :—

1. Albuminuria may occur as a transitory symptom in persons who, except for this symptom, may be judged to be perfectly healthy, for they appear to be so at the time and remain so.

2. But it may also occur in persons who, though they appear at the time to be healthy, develop signs of disease subsequently.

3. It is difficult to distinguish at a given time between those who will remain well and those who will not.

4. It is difficult to exclude for certain many of the pathological causes to which the albumin might be due. In other words, though there may be no proof that these causes are present there is equally no proof that they are absent, and in some cases the result shows that they were not absent.

5. Speaking generally, the larger the amount of albumin in the urine, and the longer it persists, the greater the probability of some permanent disease.

6. Continued observation of these cases shows that the so-called functional albuminuria does very appreciably increase the risk of the life, and that this risk grows rapidly with every year of age after 30 years. As the result of the foregoing propositions we are led to the general conclusion that functional albuminuria is never, strictly speaking, physiological at all, but that it is, on the contrary, always pathological, though not necessarily renal.

[1] Trans. Med. Soc., 1905.

